# Gap analysis of diabetes-related foot disease management systems in Pacific Islands Countries and Territories

**DOI:** 10.1186/s12913-024-10768-9

**Published:** 2024-03-11

**Authors:** Kay Y. Hon, Neil McMillan, Robert A. Fitridge

**Affiliations:** 1 Discipline of Surgical Specialties, The University of Adelaide, Adelaide, SA Australia; 2 Department of Vascular and Endovascular Surgery, Royal Adelaide Hospital, 1 Port Road, Adelaide, SA Australia; 3 Basil Hetzel Institute for Translational Health Research, The Queen Elizabeth Hospital, Adelaide, SA Australia

**Keywords:** Diabetic foot, Pacific islands, Amputation, Multi-disciplinary, Diabetes-related foot disease, Global health

## Abstract

**Background:**

Pacific Island Countries and Territories (PICTs) are known to have high prevalence of Diabetes Mellitus and high incidence of diabetes-related foot disease. Diabetes-related foot disease can lead to lower limb amputation and is associated with poor outcomes, with increased morbidity and mortality. The purpose of this study was to gain a better understanding of diabetes-related foot disease management in selected countries in PICTs and to identify potential barriers in management of diabetes-related foot disease management in the region.

**Methods:**

A cross-sectional survey was sent to eleven hospitals across six selected PICTs. The survey instrument was designed to provide an overview of diabetes-related foot disease (number of admissions, and number of lower limb amputations over 12 months) and to identify clinical services available within each institution. Two open-ended questions (free text responses) were included in the instrument to explore initiatives that have helped to improve management and treatment of diabetes-related foot diseases, as well as obstacles that clinicians have encountered in management of diabetes-related foot disease. The survey was conducted over 6 weeks.

**Results:**

Seven hospitals across four countries provided responses. Number of admissions and amputations related to diabetes-related foot disease were only reported as an estimate by clinicians. Diabetes-related foot disease was managed primarily by general medicine physician, general surgeon and/or orthopaedic surgeon in the hospitals surveyed, as there were no subspecialty services in the region. Only one hospital had access to outpatient podiatry. Common themes identified around barriers faced in management of diabetes-related foot disease by clinicians were broadly centred around resource availability, awareness and education, and professional development.

**Conclusion:**

Despite the high prevalence of diabetes-related foot disease within PICTs, there appears to be a lack of functional multi-disciplinary foot services (MDFs). To improve the outcomes for diabetes-related foot disease patients in the region, there is a need to establish functional MDFs and engage international stakeholders to provide ongoing supports in the form of education, mentoring, as well as physical resources.

**Supplementary Information:**

The online version contains supplementary material available at 10.1186/s12913-024-10768-9.

## Background

Diabetes mellitus (DM) is an increasingly prevalent condition, with up to 537 million affected individuals worldwide (1 in 11 adults) [1]. By 2045, this is projected rise to 783 million affected individuals. Over time, the incidence of DM has also increased, with one study showing a 102.9% increase from 11.3 million to 22.9 million new diagnoses from 1990 to 2017 [2]. It impacts individuals in all countries regardless of their economic state. In particular, Diabetes Mellitus (DM) is commonly cited as one of the top three deadliest non-communicable diseases in the Pacific Island Countries and Territories (PICTs) [3]. According to the IDF Atlas, six out of the top ten countries with the highest age-adjusted comparative prevalence of DM between the age of 20–79 years old can befound in this region, namely French Polynesia (25.2%), New Caledonia (23.4%), Northern Mariana Islands (23.4%), Nauru (23.4%), Marshall Islands (23.0%), and Kiribati (22.1%) [1]. These numbers are in contrast to the global overall prevalence of 10.2%, and compared to 6.4% in nearby Australia. The only country in the PICTs to have a reported prevalence of less than 10%was Samoa (9.2%). Additionally, Kiribati was ranked fourth highestin proportion of deaths under the age of 60 due to diabetes (30.4%), reflecting the high burden of DM in the country.

Diabetes-related foot disease (DRFD) is a serious and common complication of DM. It is estimated that people with diabetes have a 19–34% lifetime risk of developing a foot ulcer, which can lead to lower limb amputation if left untreated [4]. Given the high DM prevalence in the region, it is not surprising that the prevalence of diabetes-related complications, including DRFD, is higher than that reported in other regions [5]. There are limited studies on diabetes in PICTs, and even less on DRFD in the region. A systematic review reported that the prevalence of diabetes-related foot ulcer and lower limb amputations in Nauru, Solomon Islands and Vanuatu was around 5–8% and prevalence of lower limb amputation and 11% respectively [5]. A single-centre retrospective review in the Republic of Marshall Islands demonstrated that diabetes-related amputations were the fourth-most common surgical procedure performed during a 12-month period, with 4% of patients with DM during that period requiring surgical management for DRFD [6]. 

The impact of diabetes is not only significant at an individual and community level. It also carries a significant economic burden which continues to grow with the increasing prevalence of diabetes-related complications. In the PICTs, there is very limited literature to allow accurate extrapolation of the health costs associated with DRFD. Only two reviews have provided a snapshot of the associated cost of DM: in Solomon Islands, where almost 20% of total government healthcare expenditure (equivalent to A$12 million) was spent on diabetes care; and similarly in Vanuatu, where costs associated with inpatient services for diabetes-related complications made up the highest percentage of healthcare expenditure [7, 8]. Diabetes-related foot surgery was reported to be one of the costliest procedures performed in 2015, accounting for approximately 35% of total surgical expenditure in Marshall Islands [6]. These figures demonstrate the significant strain that DRFD has on already limited healthcare budgets.

Diabetes-related foot care provided by a multi-disciplinary team reduces significant complications and amputation by up to 40–60% [9]. Given the high cost associated with DRFD, it is critical that the Pacific region has the facilities and support to implement a multi-disciplinary approach to DRFD. Apart from provision of financial support, commitments to develop workforce and expertise are required to facilitate the implementation of this approach. Unfortunately, from the available data from relevant health ministries and World Health Organisations, there are significant barriers in the Pacific region to the provision of adequate staffing for multi-disciplinary teams to manage DRFD. Based on prior data, there was only a total of 35 doctors in Kiribati in 2012; only 50 physicans in Samoa in 2011 (equivalent to 4 doctors per 10,000 patients); and in Vanuatu there are only three hospitals staffed by doctors, which highlights the workforce shortage within the region [10–12]. 

There is also a lack of understanding of the current diabetes foot care models available to countries in PICTs, with very limited information available regarding the workforce that provides care to patients with DRFD in the region. The resources currently allocated are likely to be stretched by the increasing burden of DRFD. Without full understanding of the available services and supports, it would be impossible to identify and address the gaps in service delivery, and most importantly the challenges health services face in diagnosis and treating DRFD. With adequate understanding of the resources available in the region, further advocacy work can be undertaken to encourage future support for the management of DRFD in PICTs.

The purpose of this study was to survey frontline health services to gain an overview of the prevalence of DRFD and to identify challenges health services face in diagnosis and treatment of DRFD in selected PICTs, namely: Fiji, Kiribati, Samoa, Solomon Islands, Vanuatu, and Tonga.

## Methods

### Study design and participants

A mixed-methods study was conducted using a written survey, which was distributed via email to surgeon(s) in charge of each of eleven hospitals known to manage DRFD across six countries (Fiji, Kiribati, Samoa, Solomon Islands, Vanuatu, and Tonga) in the PICTs. Contact details of surgeons in charge of these hospitals were provided by Interplast Australia and New Zealand, or were sourced by contacting local health services.

### Survey

The survey instrument (Supplementary 1) was designed by the authors for the study. The survey aimed to ascertain the capacity of each hospital, and numbers of admissions and procedures relating to DRFD. To understand the challenges in diagnosing and treating DRFD in PICTs, data were collected to determine clinical services available in the facility to provide multidisciplinary foot care, and to ascertain the potential deficits in services required for multidisciplinary foot care. Two open and exploratory questions were included to enquire on initiatives that have helped to improve management and treatment of DRFD, and barriers faced in management of DRFD. Free text response feedback was analysed qualitatively using thematic analysis.

The survey period was conducted over 6 weeks and reminder emails were sent every fortnight. Phone calls were made to hospitals that did not respond as an attempt to organise phone interview to complete the survey.

## Results

Seven out of the eleven hospitals across six countries contacted responded to the survey. No response was received from any hospitals in Vanuatu and Tonga.

Numbers of amputations and DRFD admissions were reported as an estimate by clinicians in Table 1. The highest number of inpatient admissions for DRFD were reported by Colonial War Memorial Hospital in Fiji with approximately 1200 admissions over 12 months, followed by Labasa hospital in Fiji (800 admissions) and National Referral Hospital (NRH) in Solomon Island (300 admissions).

**Table 1:** An overview of diabetes-related foot disease in various institutions

	Population	Number of beds	Admissions for DRFD	Number of lower limb amputations	
				Minor	Major
**Fiji**					
Labasa Hospital	140,000	130	800	40	30
Latouka Hospital	400,000	350	N/A	N/A	N/A
Colonial War Memorial Hospital	500,000	500	1200	195	251
**Kiribati**					
Tungaru Central Hospital	100,000	140	90	48	64
**Samoa**					
Malietoa Tanumafill II Hospital	18,255	29	184	3	7
**Solomon Islands**					
Gizo Hospital	70,000	96	57	13	6
National Referral Hospital	200,000	360	300	N/A	68

As summarised in Table 2, DRFD was primarily managed in the region by general physicians, general surgeons, and orthopaedic surgeons in the hospitals surveyed, as there were no subspecialty services (vascular surgery, endocrinology, renal medicine, or infectious diseases).

**Table 2:** Access to specialty services at each institution

	Fiji			Kiribati	Samoa	Solomon Island	
Hospital	Labasa Hospital	Latouka Hospital	Colonial War Memorial Hospital	Tungaru Central Hospital	Malietoa Tanumafill II Hospital	Gizo Hospital	National Referral Hospital
General physician	8	5	Yes	Yes	2	1	5
Endocrinology	0	0	0	0	0	0	0
Infectious diseases	0	0	0	0	0	0	0
Renal physician	0	0	0	0	0	0	0
General Surgery	10	5	Yes	Yes	0	1	3
Orthopaedic	3	4	Yes	0	0	0	3
Nurse	200	Yes	Yes	Yes	65	Yes	30
Podiatry	0	0	0	0	0	0	0
Physiotherapy	7	5	Yes	Yes	0	1	3
Orthotics & Prosthetics	0	0	0	Yes	0	0	0
Occupational therapy	0	0	0	0	0	2	0
Radiology	15	2	0	0	2	4	3
Diabetic foot clinic	Yes	Yes	Yes	0	0	0	4
Community nursing	100	0	Yes	N/A	10	0	N/A

Data presented as number of staff, yes if services available but unclear number of staff or N/A if no response received

All three hospitals in Fiji surveyed had dedicated footcare clinics with support from Diabetes Fiji, a member of the International Diabetes Federation. However, there was no podiatrist nor orthotist support in any of these clinics. Tungaru Central Hospital in Kiribati was the only institution surveyed with access to orthotist and prosthetist services, who stated that a diabetes foot clinic was maintained, but provided no details on its setup and management. NRH in Solomon Islands also managed four diabetes foot clinics in the region, chiefly run by senior nurses. There was no access to inpatient podiatry services in the region reported.

Imaging services were also limited in the region (Table 3). All hospitals had access to plain radiography. However, none reported access to vascular imaging (vascular ultrasound, computed tomography (CT) Angiography and digital subtraction angiography). Magnetic resonance imaging (MRI) was available in Fiji. However, patients from peripheral hospitals must travel up to 215 km via air or sea to the capital city, Suva, located on the island of Viti Levu. It can take up to two weeks for these arrangements to be made.

**Table 3:** Access to imaging services at each institution

	Fiji			Kiribati	Samoa	Solomon Island	
Hospital	Labasa Hospital	Latouka Hospital	Colonial War Memorial Hospital	Tungaru Central Hospital	Malietoa Tanumafill II Hospital	Gizo Hospital	National Referral Hospital
Plain radiography	1 h	30 min	Yes	Yes	2 h	Yes	1
Magnetic resonance imaging (MRI)	5–14 days *	No	Yes	No	No	No	No
Nuclear imaging	No	No	No	No	No	No	No
Ultrasound	1–5 days	1 day	No	No	1 day	Yes	No
Vascular ultrasound	No	No	No	No	No	No	No
Computed tomography angiogram (CT-A)	1 day to 1 month	No	No	No	No	No	No
Digital Subtraction angiography	No	No	No	No	No	No	No

Data presented as number of staff, yes if services available but unclear number of staff or N/A if no response received

All hospitals were able to perform complete blood counts, but access to biochemistry testing and histopathology was limited to some hospitals (Table 4). In Fiji, histopathology testing could only be carried out at Colonial War Memorial Hospital, which is at least 4 hours’ drive (216 km) from Lautoka and Labasa Hospital and requires approximately one month to process. The MTII in Samoa only had facilities to perform complete blood counts; other blood tests, including multiple biochemical analysis (MBA+), required referral to Tupua Tamasese Maeole Hospital (TTMH) on the main island, Uplou. There were only complete blood counts available at Gizo Hospital in Solomon Islands, whilst histopathology results could only be obtained after several weeks following processing by Pathology Queensland (Queensland, Australia).

**Table 4:** Pathology services available to each institution

	Fiji			Kiribati	Samoa	Solomon Island	
Hospital	Labasa Hospital	Latouka Hospital	Colonial War Memorial Hospital	Tungaru Central Hospital	Malietoa Tanumafill II Hospital	Gizo Hospital	National Referral Hospital
Complete blood counts	6 h	1 h	N/A	N/A	24 h	hours	1 day
Multiple biochemical analysis	12 h	1 h	N/A	N/A	N/A	N/A	1 day
HbA1c	12 h	1 h	N/A	N/A	N/A	N/A	1 day
Microscopy, culture and sensitivity	2–5 days	2 days	Yes	Yes	N/A	N/A	4 days
Histopathology	2–6 weeks	4 weeks	N/A	N/A	N/A	weeks	21 days

Data presented as turnaround time or Yes/No to indicate if available or unavailability of services, or N/A if no response received

Common themes identified in the barriers faced in management of DRFD by clinicians were broadly centred around resource availability, community awareness and education, and professional development.

A recurring theme identified was the lack of resources to provide evidence-based care for affected patients as presenting the most significant barrier clinicians faced in management of DRFD. There was a lack of access to (1) specialised dressings (e.g., negative pressure wound therapy), (2) clinical specialties such as podiatry, endocrinology, infectious disease, interventional radiology and vascular surgery services, (3) appropriate diagnostic tools such as vascular imaging,; and (4) on-site biochemistry testing. The limited availability of these clinical services presents a major challenge in establishing a functional, multi-disciplinary high-risk foot care team. One hospital identified the greatest needs to improve DRFD outcomes as “having infrastructure, including but not limited to a physical space specifically for diabetic foot with a trained team and surplus of clinical supplies”. Two hospitals also identified inadequate “supply of drugs and wound dressings to rural clinics” as a barrier to providing adequate care for patients with DRFD.

Gizo Hospital noted that improvement in staffing in the hospital, with senior staff being involved in the care of DRFD, and adopting a multi-disciplinary approach were the most significant steps in helping to improve DRFD care in their patient population. Previously, the hospital had been staffed by junior registrars. However, since 2020, a general surgeon and physician had also been appointed. Senior nurses had also received training in wound care management. The hospital also had a well-established physiotherapy department which recruited community rehabilitation officers to help support patients after discharge. Additionally, there also had been an increase in anaesthetic support in the hospital, with registrars proficient in administering regional and general anaesthesia being posted to the hospital.

Our survey responses emphasised the need to enhance awareness about the impact of diabetes as a disease, and to educate the public about the importance of lifestyle changes to prevent DRFDs. Lack of adherence with medical advice and wound care instruction was identified as a significant issue that hinders outcomes for patients with DRFDs. Some hospitals found local community health and public health initiatives valuable. One of the programs specifically identified was the *Package of Essentials NCD (PEN) Fa’asamoa*, which is an initiative working closely with villages and primary health centres for early diagnosis and referral of noncommunicable diseases, of which DM is of one of the priority areas [13]. 

Education and training were also highlighted as areas for improvement, including (1) providing ongoing training and up-to-date knowledge in identification, initial management, and prompt referral of DRFD to referral hospitals, (2) understanding the importance of optimal blood glucose level and blood pressure management in patients with DRFD, and (3) providing continuous education to nurses based in rural or remote areas to support and empower them to manage DRFD. A general surgeon managing nearly 800 cases of DRFD each year advocated for the development of culturally- and geographically-appropriate clinical guidelines to empower healthcare providers to “be more aggressive in active intervention of diabetic foot sepsis at earlier opportunity”.

## Discussion

For health services to be effective in improving the outcomes of diabetes-related foot disease in the region, there is a need to act on the identified gaps in human, technical, and clinical resources.

From this brief survey, it is clear that major issues relating to the care of DRFD in the region include the lack of physical and human resources. This was an observation also made by Tin and colleagues that a lack of both resources and the distribution thereof have negatively impacted DRFD service delivery in the region [5]. There is limited access to specialists and multi-disciplinary expertise that are central to improving the outcomes of DRFD. Most importantly, while PICTs currently do not have enough health workers to adequately service their populations, their health sector budgets cannot support the creation of more posts. It has been noted that “the Pacific Islands are a chronically medically-underserved region” with limited resources to train and retain health professionals [14]. The survey respondents also noted a significant deficit in sustainable programs and partnerships with stakeholders in the management of DRFDs, as most partnerships have funding arrangements delivering only short-term activities.

There was also a large variation in staffing levels between hospitals servicing similar populations, resulting in lack of availability of specialised services in centres that manage DRFD, and a geographical imbalance in the distribution of the health workforces, as seen in Fiji where the larger Lautoka Hospital has fewer physicians compared to the smaller Labasa hospital. This confirmed the need to train and appoint healthcare providers with the necessary skills and experience in the right location, which was one of the key strategies outlined in the Pacific Heads of Health Meeting in 2018 [15]. 

The biggest identified barrier for care of DRFD was the availability of resources to provide evidence-based care for affected patients. There was most notably a need for improved capacity to allow institutions to perform on-site biochemistry testing and testing for microscopy, culture, and sensitivity (MCS) of wound specimens. On-site biochemistry testing would allow assessment of haemoglobin A1c (HbA1c), which is an important test to examine the adequacy of diabetes management, determining optimal diabetic control is essential in healing of diabetes-related foot ulcers. Additionally, the use of results from MCS to tailor antimicrobial therapy is a central recommendation of the International Working Group on the Diabetic foot (IWGDF). Improving the supply of medications and wound dressings to rural clinics would also likely improve the outcomes for patients with DRFD. Finally, encouraging the use of clinical photography and provision of adequate instruments to allow high-quality photographs to be taken have also been identified as an area of opportunity. The use of photographs in clinical practice can assist in monitoring wound progression and to help evaluate effectiveness of treatment. It can also be used as part of telehealth for disparate communities.

The lack of Allied Health (AH) input in the management of DRFD is cause for concern, as routine podiatry care and adequate offloading of foot pressure areas are important in the management of DRFD [16]. It is likely that non-AH clinicians would have to assume these management roles through task shifting. Task-shifting is a structure that is very evident in, and the essential core of, the diabetes foot care model in the region– where both nursing and medical staff have taken on tasks that otherwise would have been allocated to specialised AH staff in major centres. Task-shifting is a practical and potentially cost-saving approach to increase the number of services available in the short term [17, 18]. However, improved AH access is needed to improve patient outcomes in the long term, such as a partnership to develop a formal education or professional development program in the region to facilitate upskilling of nursing and medical staff to better care for patients with DRFD.

The biggest barrier for our survey was limited engagement from clinicians. Multiple attempts via email correspondence and phone calls to contact relevant clinicians and administrative staff were unsuccessful in many cases. The lack of engagement should not be considered as a reflection of lack of interest or effort, but rather should be included as a learning opportunity from this research. Evaluation should be undertaken to assess the factors that lead to lack of engagement– whether the survey method was appropriate, if the clinicians have access to facilities to complete the survey, if the clinicians have the data required to complete the survey, and what would have been the preferred modality to connect with them.

The lack of functioning data management systems contributed to some difficulty in obtaining accurate data for this research. Almost the entirety of our results were based on estimation by contactable clinicians, and results need to be interpreted with caution. This was also reported in a clinical audit that identified a deficiency in access to accurate data with regard to prevalence of DM in PICTs, which likely resulted in underreporting the prevalence of DM [19]. Reviews by Gardner et al. and Hoyt et al. have already highlighted that inadequate data collection and lack of functioning digital health solutions are obstacles to any primary care quality improvement programs in the region [20, 21]. Unsuccessful attempts were made to contact respective ministries of health and hospital management to obtain annual health status reports to determine relevant data, which is also likely a reflection of inadequate data management and distribution.

Attempts to characterise and identify the healthcare workforce in PICTs is challenging. This literature review was limited by paucity of data available and outdated grey literature [22–24]. Distribution of surveys was challenged by difficulty in establishing reliable contacts. Ongoing collaboration between investigators and relevant health information systems is needed to promote data sharing, synthesis, and analysis in order to gain an accurate picture of DRFD in PICTs. Success has also been previously found with surveys completed by academics visiting the regions. For example, Wiegmann et al. were able to successfully collect data on cost and benefit of diabetic foot clinics via in-person data collection and interviews, including data on surgeries performed [25]. Their approach of having the researcher on site to obtain data was successful and should be considered as the ideal solution for future research.

The impact of COVID-19 pandemic in the region must also be considered. In early 2020, each of the PICTs implemented strict border closures and lockdown to prevent transmission of COVID-19 due to the vulnerability of their individual communities. A consequence of these restrictions was an increased workload required to sustain the already-strained systems to provide optimal routine care, likely restraining quality improvement investigations and initiatives. Additionally, due to the border closures, many volunteers were unable to travel to the region to provide clinical, education, and administrative support.

Beyond the scope of the survey, there are two important barriers that are likely to influence outcomes for patients with DRFD: (1) the accessibility and cost of medications to treat diabetes and diabetes-related foot infection, and (2) the sufficient availability of well-established and funded primary care services that can diagnose and manage diabetes and promptly refer patients to multi-disciplinary foot services when clinically indicated. Both are critical components in the slow advancement of Universal Health Care (UHC) in the PICTs, which is at the core of the World Health Organisation’s Healthy Island vision. Access to essential medicines and provision of primary health care in PICTs can be limited due to the challenges with geographical remoteness and the unique healthcare needs of the local population. In 2022–2023, hospitals and primary health services in Solomon Islands faced critical medical supply shortages, which has undoubtedly put further strain on their health system in provision of optimal care to patients [26]. There are significant challenges in ensuring availability and compatibility of health workers to provide continuity of care especially in primary care level, due to health workforces and infrastructure constraints, with significant disparities noted between the urban areas and the remote islands in the region [27]. Vanuatu, Kiribati Samoa and Solomon Islands in particular have been identified in the WHO health workforce support and safeguard list due to low density of health professionals below the median required for UHC [28]. These primary health system challenges no doubt have profound downstream impacts on DRFD management and tertiary care.

The main limitation of this study was selection bias of respondents, as well as potential issues with response accuracy, leading to likely underestimation of the burden of the DRFD in the region and its effects on patients’ outcomes. This highlights the notable scarcity of research, which restricts the understanding of and capacity to address challenges in management of DRFD in the region. Nevertheless, our study was able to provide an overview of services available in PICT for management of DRFD and provided a unique insight into the barriers many healthcare providers faced in their endeavour to improve outcomes for their patients suffering with DRFD.

## Conclusion

Our survey highlights the critical gaps in care and resources for treating diabetes-related foot disease in the PICTs. These gaps are likely to exacerbate the burden of DRFD in the region– including the physiological and psychological impact of suffering for patients from DRFD on a personal level, the need for carers for those with DRFD on a community level, and the macroeconomic impact from increasing health expenditures and lost national productivity. There is a need for longitudinal cohort studies and epidemiological research to better understand the prevalence and incidence of DRFD in the region. The region needs strong advocacy for support and change implementation in culturally-appropriate models of diabetes foot service delivery to improve the outcomes for patients with DRFD in PICTs.

### Electronic supplementary material

Below is the link to the electronic supplementary material.

Supplementary Material 1

## Data Availability

The data used and/or analysed during the current study are available from the corresponding author on reasonable request.

## Abbreviations

DM: Diabetes mellitus
DRFD: Diabetes-related foot disease
IWGDF: International Working Group on Diabetic Foot
MCS: Microscopy, culture and sensitivity
MDF: Multi-disciplinary foot service
PICTs: Pacific Island Countries and Territories
