# Supplementary material for: Gap analysis of diabetes-related foot disease management systems in Pacific Islands Countries and Territories
Source: BMC Health Serv Res. 2024 Mar 11;24:324. doi: 10.1186/s12913-024-10768-9 (PMC10929083; doi:10.1186/s12913-024-10768-9)
Supplement: Supplementary file 1 — Supplementary Material 1 [file 12913_2024_10768_MOESM1_ESM.docx]

**Supplementary 1: Survey instrument developed and used to provide an overview of diabetes-related foot disease and to identify clinical services available within each institution.**

**About your hospital:**

| What are the total number of beds in your hospital? |  |
| --- | --- |
| What is the catchment population of your hospital? |  |
| What were the number of admissions for diabetes-related foot disease in 2021? |  |
| How many major amputation (above ankle) were performed in 2021? |  |
| How many minor amputation (below ankle) were performed in 2021? |  |
| Under which team are patients with diabetes related foot disease admitted? |  |
| Is there an electronic health record available for your hospital? |  |
| If yes to above, what is the system called? |  |

**Clinical services:**

Specialty medicine and services

| What specialty services does your institution has access to? | Number of staff |
| --- | --- |
| General physician |  |
| Endocrinology |  |
| Infectious diseases |  |
| Renal physician |  |
| General surgery |  |
| Orthopaedic surgery |  |
| Nurse |  |
| Podiatry |  |
| Physiotherapy |  |
| Orthotics and prosthetic |  |
| Occupational therapy |  |
| Radiology |  |
| Diabetic foot clinic |  |
| District/community nursing care |  |

| For imaging services that are not available at your institution, where would you refer your patient? |
| --- |
|  |

Imaging services:

| Which of the following imaging services does your institution have access to? | | Turnaround time  (hour or day) |
| --- | --- | --- |
|  | Plain radiography (X-ray) |  |
|  | Magnetic resonance imaging (MRI) |  |
|  | Nuclear imaging such as bone scan |  |
|  | Ultrasound (vascular imaging) |  |
|  | Computed tomography Angiogram (CT-A) |  |
|  | Digital subtraction angiography |  |
|  | Others: |  |

| For imaging services that are not available at your institution, where would you refer your patient? |
| --- |
|  |

Pathology services :

| Which of the following pathology services does your institution have access to? | | Turnaround time  (hour or day) |
| --- | --- | --- |
|  | Complete blood counts |  |
|  | Multiple biochemical analysis (Electrolytes, Renal and kidney function) |  |
|  | HbA1c |  |
|  | Microscopy, culture and sensitivity (MCS) |  |
|  | Histopathology |  |

| For pathology services that are not available at your institution, where would you refer your patient? |  |
| --- | --- |

| What would you consider your greatest need(s) to improve diabetes-related foot disease outcomes? |
| --- |
|  |
| What would you consider the most significant change/initiatve(s) in your institution that has helped to improve diabetes-related foot disease outcomes? |
|  |

**Barriers and improvements**

| We are hoping to undertake further studies in the region to evaluate prevalence of diabetes and diabetes related foot disease (number of patients with diabetes, number of patients with diabetes-related foot disease being seen in outpatients or inpatients, number of admissions for diabetic foot related diseases and number of amputations due to diabetes-related foot disease).  Can you let us know who to contact or which institution would be able to provide this information?. |
| --- |
|  |
